# Alterations of microbiota in urine from women with interstitial cystitis

**DOI:** 10.1186/1471-2180-12-205

**Published:** 2012-09-13

**Authors:** Huma Siddiqui, Karin Lagesen, Alexander J Nederbragt, Stig L Jeansson, Kjetill S Jakobsen

**Affiliations:** 1 Department of Biology, Centre for Ecological and Evolutionary Synthesis (CEES), University of Oslo, P.O. Box 1066, Blindern, 0316, Oslo, Norway; 2 University Hospital HF Aker-Oslo and Faculty of Medicine, Division of Medicine, ME/CFS-center, University of Oslo, P.O. Box 4956, Nydalen, 0424, Oslo, Norway

## Abstract

**Background:**

Interstitial Cystitis (IC) is a chronic inflammatory condition of the bladder with unknown etiology. The aim of this study was to characterize the microbial community present in the urine from IC female patients by 454 high throughput sequencing of the 16S variable regions V1V2 and V6. The taxonomical composition, richness and diversity of the IC microbiota were determined and compared to the microbial profile of asymptomatic healthy female (HF) urine.

**Results:**

The composition and distribution of bacterial sequences differed between the urine microbiota of IC patients and HFs. Reduced sequence richness and diversity were found in IC patient urine, and a significant difference in the community structure of IC urine in relation to HF urine was observed. More than 90% of the IC sequence reads were identified as belonging to the bacterial genus *Lactobacillus*, a marked increase compared to 60% in HF urine.

**Conclusion:**

The 16S rDNA sequence data demonstrates a shift in the composition of the bacterial community in IC urine. The reduced microbial diversity and richness is accompanied by a higher abundance of the bacterial genus *Lactobacillus*, compared to HF urine. This study demonstrates that high throughput sequencing analysis of urine microbiota in IC patients is a powerful tool towards a better understanding of this enigmatic disease.

## Background

Interstitial Cystitis or Painful Bladder Syndrome (IC/PBS) is a chronic condition characterized by frequent urination and bladder pain, which often results in reduced quality of life. Clinicians experience that this disease is becoming more prevalent [1]. While evidence suggests that about 90% of those affected are female, some urologists consider chronic bacterial prostatitis to be the male equivalent of IC.

The traditional definition of this illness is chronic sterile bladder inflammation of unknown etiology and it has not been possible to prove any causative pathogenic agent for this syndrome [2,3]. Currently there are four major hypotheses of pathogenesis: 1) autoimmunity, 2) deficiency of the glycosaminoglycan layer causing increased bladder wall permeability, 3) neurogenic inflammation and 4) chronic infection [4].

While several features of IC have suggested an infective etiology, numerous studies using traditional culture techniques have failed to provide consistent evidence that IC is associated with infection. It has been proposed that possible microbial agents causing this disease could be difficult to cultivate or are present in numbers too low to be confirmed in the laboratory [5]. Advances in molecular-based diagnostics have made it possible to overcome the limitations of culture-based detection. Investigators have used PCR, cloning and 16S ribosomal DNA (rDNA) sequencing to search for pathogenic agents in bladder biopsies and urine specimens of IC patients [6-11], but with conflicting results. However, some of these studies have indicated that women with IC may have a higher prevalence of bacteria in the urine than those without IC [6,8,9].

Furthermore, clinical studies have demonstrated that administration of antibiotics may sometimes be correlated with decreased symptoms in patients [12-14]. This can be due to both inhibition of bacterial growth or as a conventional anti-inflammatory effect of doxycycline. A study by Zhang *et al*. (2010) [15] not only demonstrates improvement in symptoms, but also a decreased level of nanobacteria after antibiotic treatment, strongly suggesting a microbial association of IC in some cases.

We recently developed approaches to assess the major microbial populations in female human urine, based on 16S rDNA PCR followed by 454 pyrosequencing and analyses using a suite of bioinformatics tools (Siddiqui *et al*. ( [2011]) 0 [16]. We have shown that healthy female (HF) urine is a complex milieu with many different bacteria present. The normal human urine microbiota includes numerous fastidious and anaerobic microbes, which are potentially pathogenic [16-19]. In this work we applied these techniques in a prospective study to describe the microbial community present in urine from IC patients. We also performed a comparative analysis between the IC sequence dataset and the HF dataset previously generated [16] to determine to what extent the bacterial profiles differ. Our analyses indicate important differences between the two microbiota. We observe a lower complexity and variation between urine from IC individuals in relation to HF individuals.

## Methods

### Urine sampling

This study was approved by the Regional Committee for Medical Research Ethics East –Norway (REK Øst Prosjekt 110-08141c 1.2008.367), and the samples were taken with informed consent. 8 female patients of age from 27 to 67 years (P1 = 59, P2 = 40, P3 = 27, P4 = 47, P5 = 31, P6 = 35, P7 = 32, P8 = 67) underwent thorough clinical examination including cystoscopy and fulfilled the criteria of European Society for the Study of Interstitial Cystitis (ESSIC) [20]. All patients had an established diagnosis of IC for more than four years. Midstream urine (30 ml) was collected by the clean catch method with labial separation supervised by an urotherapy nurse. Specimens were kept at 4°C, and within an hour processed for DNA isolation. All specimens used were culture-negative, as tested by the Urological Clinic at the University Hospital HF Aker-Oslo. None of the patients was receiving antibiotics at the time samples were taken, nor prior to that according to hospital records.

### Sample processing and DNA isolation

Sample processing and DNA extraction was performed as previously described in Siddiqui *et al*. (2011) [16]. Briefly, urine aliquots (30 ml) were pelleted by centrifugation and total DNA was isolated from sediments using DNeasy Blood & Tissue kit (QIAGEN, Germany), preceded by incubation with POWERlyse (lysis buffer) (NorDiag ASA, Oslo, Norway). Finally, the DNA was eluted in 100 μl of AE buffer from the kit. The DNA concentrations in the samples (P1-P8) were measured by Quant-iT PicoGreeen dsDNA assay kit (Molecular Probes, Invitrogen USA) and ranged from 0.22 ng/μl to 4.36 ng/μl.

### 16S rDNA PCR and 454-pyrosequencing

For each IC urine sample, we amplified 16S rDNA sequences using two different primer sets specific for the V1V2 and V6 hypervariable regions followed by 454 pyrosequencing as described in Siddiqui *et al*. (2011) [16]. Each of the primers consisted of a target specific region at their 3’ end (V1V2 or V6) and an adapter sequence (Primer A or Primer B) at their 5’ end as needed for GS FLX amplicon sequencing (454 Life Sciences, USA). Equal amounts of the two different amplicons (both V1V2- and V6-region) for a single subject were pooled and sequenced using GS-FLX chemistry in the same lane of a Pico-Titer plate divided into 16 lanes, except for samples P1, P2 and, P3, for which each amplicon (V1V2 and V6) was sequenced in a separate lane. 454 pyrosequencing was performed by the Norwegian Sequencing Centre (NSC) at the Department of Biology, University of Oslo, Norway.

### Sequence read preprocessing

Sequence read preprocessing was done as described in Siddiqui *et al*. (2011) [16]. In brief, a total of 187,901 reads were produced from IC female urine samples. The initial sequence reads were split into two pools using the V1V2 and V6 primer sequences via the sfffile program from 454 Life Sciences (Roche), thus reducing the sequences to 172,931 IC urine reads (Table 1) due to the program splitting on an exact primer match. With a minimum length cutoff of 218 and 235 nt for the V1V2- and V6-regions, respectively, the sequences were processed through the Pyronoise program [21] to reduce possible insertion/deletion errors at homopolymer runs. After denoising using Pyronoise, one sequence per cluster is retained together with the number of total reads mapping to that cluster.

**Table 1:** Sampling depth and biodiversity found by amplicon 454 pyrosequencing V1V2 and V6 region from urine

		***Combined sequence pool from HF urine***^***1***^		***Combined sequence pool from IC urine***^***2***^	
		**V1V2**	**V6**	**V1V2**	**V6**
***Preprocessing***					
	Total reads	78346	74067	74211	98720
	Length cutoff ^3^	48861	45382	46272	62325
	Denoised^4^	48860	45136	46267	62173
	Cleaned^5^	48452	44760	46138	62032
***Taxonomy analysis***					
	Phyla^6^	10	8	5	7
	Genera^6^	35	28	23	25
***OTU and Diversity indices***					
	Cleaned^5^	48452	44760	46138	62032
	Silva 16S alignment^7^	46001	44146	44594	61170
	Unique OTUs	974	2045	514	1432
	OTUs^8^ (3%)	724	1537	344	1008
	OTUs^8^ (6%)	615	1265	292	786
	Chao1^9^ (3%)	1435	3936	357	2485
	Chao1 LCI95	1261	3521	675	2172
	Caho1 HCI95	1664	4437	1137	2883
	Shannon index^10^ (3%)	2.62	3.02	1.67	1.95
	Inverse Simpson index^11^ (3%)	6.97	7.03	3.57	3.72

^1^Combined sequence data from eight healthy female (HF) urine samples, sequences generated in prior study (Siddiqui *et al*. (2011) [16]).

^2^Combined sequence data from eight interstitial cystitis (IC) urine samples.

^3^Length cutoff at minimum 218 nt for V1V2 and 235 nt for V6 reads.

^4^Total number of sequences after processing the dataset through Pyronoise [21].

^5^The number of reads per dataset after removal of sequences that could be from the same source as those in the contamination control dataset as described in Siddiqui *et al*. (2011) [16].

^6^Number of phyla and genera based on taxonomic classification by MEGAN V3.4 [23,24].

^7^The number of total reads after Silva 16S alignment as recommended by MOTHUR [29].

^8^OTUs: Operational Taxonomic Units at 3% or 6% nucleotide difference.

^9^Chao1 is an estimator of the minimum richness and is based on the number of rare OTUs (singletons and doublets) within a sample.

^10^The Shannon index combines estimates of richness (total number of OTUs) and evenness (relative abundance).

^11^Inverse Simpson index (1/D) is an indication of the richness a community with uniform evenness would have at the same level of diversity. It takes into account the number of OTUs present, as well as the abundance of each OTU.

The bacterial identification technique of broad range 16S rDNA PCR is highly sensitive to environmental contamination. To control for this the IC urine sample sequence sets were stripped for sequences that could stem from contamination sources. This was done by using contamination control sequences (total = 25,246) from negative control extractions (buffer and kit reagents) followed by PCR and pyrosequencing, as reported in Siddiqui *et al*. (2011) [16]. A complete linkage clustering at 1% genetic difference of each sample together with its respective control was performed using ESPRIT ( http://www.biotech.ufl.edu/people/sun/esprit.html[22]). Any sample sequences found in clusters where ≥ 50% of the sequences belonged to the contamination control were excluded from subsequent analyses (details in Siddiqui *et al*. (2011) [16]).

Sequence data generated in this study were submitted to the Sequence Read Archive with the study accession number ERP001705. The dataset is available at http://www.ebi.ac.uk/ena/data/view/ERP001705.

### Taxonomical analysis

For taxonomic grouping of the sequence reads, MEGAN V3.4 http://www-ab.informatik.uni-tuebingen.de/software/megan/welcome.html[23,24] was used. First, the sequence reads were compared to a curated version of the SSUrdp database [25] using blastn with a maximum expectation value (E) of 10^-5^. To reflect the actual abundance behind every denoised sequence cluster, each entry in the blast result file was replicated as many times as the total number of reads that mapped to that query sequence (for detailed procedure and parameters see Siddiqui *et al*. (2011) [16]). When comparing the individual datasets using MEGAN, numbers of reads were normalized up to 100,000 for every dataset.

Metastats, statistical methods ( http://metastats.cbcb.umd.edu/, [26,27]) for detecting differentially abundant taxa, was used to reveal significant differences between IC urine microbiota and HF urine microbiota (taxonomy assessed in Siddiqui *et al*. 2011 [16]). This method employs a false discovery rate to improve specificity in high-complexity environments, and in addition handles sparsely sampled features using Fisher’s exact test. The Metastats *p* - values at different taxon levels, which were assigned using MEGAN, are listed in Additional file 1: Table S1. A *p* - value ≤ 0.05 was considered significant.

### Comparative OTU based clustering analysis of IC and HF urine

Numbers of operational taxonomical units (OTUs), rarefaction curves and diversity indices were calculated using MOTHUR v1.22.2 [28,29] (see Table 1). To enable comparisons, the HF sequences generated in Siddiqui *et al*. (2011) [16] were reanalyzed along with the IC dataset from this study. Briefly, the sequences were aligned to the Silva 16S alignment as recommended by MOTHUR [29] – sequences not aligned or aligned outside of where 95% of all of the sequences aligned were removed from the datasets. For an improved OTU clustering single linkage preclustering [30] was performed, allowing two nucleotides to differ between sequences, before clustering using average linkage. The processing was done both on each separate sample and on pooled V1V2 and V6 sequences for both IC and HF samples. We also calculated the OTUs and Shannon index for normalized numbers of sequences for each separate sample [31]. A random number of reads, corresponding to the lowest number of sequences in a sample group, i.e. 2,720 for V1V2 and 2,988 for V6, was picked 100 times from each sequence set. These new sequence sets were processed through MOTHUR in the same fashion as the full sequence sets and the average of the resulting OTUs and Shannon values are shown in Additional file 2: Table S2.

The differences in Shannon indices and inverse Simpson indices for the two communities (IC and HF urine) were also statistically evaluated by Wilcox rank sum test in R, for both the V1V2 and V6 amplicon datasets.

Venn diagrams were generated for both data sets using MOTHUR to calculate how many OTUs were shared between the two communities. To further explore the relationships between the two microbial communities, samples were clustered into Newick-formatted trees (using the UPGMA algorithm) with distance between communities calculated with θYC coefficient as a measurement of dissimilarity between community structures [32] in MOTHUR. In addition, weighted UniFrac testing [33] was performed to determine the statistical significance of clustering within the tree. A non-metric multidimensional scaling (NMDS) plot was generated in R for the distances calculated using θYC measures for each sequence dataset (V1V2 and V6), knowing that θYC weighs rare and abundant OTUs more evenly than other metrics such as Jaccard.

## Results

### 454 pyrosequenced 16S rDNA amplicon sequences

After preprocessing of the raw IC 454 reads as described in Siddiqui *et al*. (2011) [16], we obtained a total of 46, 138 and 62,032 16S rDNA sequences for V1V2 and V6 regions, respectively, see Table 1. For comparison purposes, the preprocessing information for the HF urine sequences reported in Siddiqui *et al*. (2011) [16] is also listed in the table. Average number of reads per IC sample was 5,767 and 7,754 for V1V2 and V6, respectively (range: V1V2 3035–9506; V6 4900–14602) see Additional file 2: Table S2. 97% of the preprocessed sequences were classified to phylum, order and family level, and 95% of the sequences were identified down to genus level.

### Composition of the IC urine microbiota

**Figure 1:**
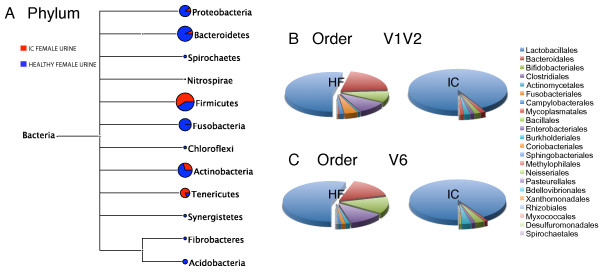
**Summary of the microbial phyla and orders detected in interstitial cystitis urine and healthy female urine. A**: A comparative taxonomic tree view of 16S rDNA sequences from interstitial cystitis (**IC**) urine and healthy female (**HF**) urine assigned to the phylum level as computed using MEGAN V3.4. Normalized counts by pooling together results from V1V2 and V6 16S rDNA sequence datasets were used for both IC and HF urine. **B** and **C**: Comparison of taxonomic assignments for IC and HF urine sequences at the order level, showing an increase of the order *Lactobacillales* in IC urine sequences relative to HF urine, for both V1V2 (**B**) and V6 datasets (**C**).

In total, 7 phyla were identified by the 16S rDNA sequences when the two different amplicon libraries (i.e.V1V2 and V6 16S regions) were considered together (Figure 1A). 93% of the bacterial DNA sequences were assigned to *Firmicutes,* while the other 7% were assigned to 6 additional phyla. *Actinobacteria* was the second major phylum with 5% of the sequence abundance. *Bacteroidetes* and *Tenericutes* were represented by 1% of total bacterial sequences each, while three phyla – *Proteobacteria*, *Fusobacteria* and *Nitrospirae* – were detected by less than 1% of the assigned sequences.

In comparison to HF urine (Siddiqui *et al*. (2011) [16]), IC urine has a significantly higher proportion of *Firmicutes* (*p* ≤ 0.05, *p* value from Metastats for V1V2) (65% vs 93%, respectively) and reduced proportions of the other 5 common phyla (Figure 1A). Interestingly, the phylum *Nitrospirae* was only detected in IC urine. Five additional phyla present in HF urine (Siddiqui *et al*. (2011) [16]) were not identified in IC urine at all (Figure 1A). The distribution of major phyla in IC urine was similar for both the V1V2 and V6 sequence dataset, although *Fusobacteria* and *Nitrospirae* were only identified by the V6 sequence dataset.

Sequence reads for all phyla but one (*Nitrospirae* 0.003% of the reads) were further classified to order level. 16 of the 22 orders identified in healthy urine (Siddiqui *et al*. (2011) [16]) were also detected in IC urine. A significant shift in the bacterial composition was observed as a result of an increase of *Lactobacillales* (Figure 1B and C) (*p* ≤ 0.05, *p* value from Metastats for V1V2) in the IC urine microbial community relative to HF urine. 92% and 91% of the reads for V1V2 and V6 respectively, were assigned to this order. In HF urine only 53% of the reads for V1V2 and 55% for V6 were assigned to *Lactobacillales*. The abundance of other major orders seen in HF urine is reduced in IC samples (Figure 1B and Additional file 1: Table S1).

All sequence reads assigned to the order level were additionally assigned to family level. Among the 26 families identified, only 21 were assigned to different genera. Four of those families that were not further assigned (*Pasteurelacae, Neisseriacae, Methyliphilaceae,* and *Micrococcaceae*) were also detected in the HF urine study. *Saprospiraceae*, on the other hand was only found in IC urine.

At the genus level, the pooled sequences were assigned to 31 different genera, with 23 and 25 different genera for V1V2 and V6 analysis, respectively. *Lactobacillus* was the most abundant genus in both datasets and comprised a total of 92% of the sequences. *Gardnerella* and *Corynebacterium* were the two other major genera identified with 2% sequence abundance each. *Prevotella* and *Ureaplasma* were each represented by 1% of the sequences assigned. The other 26 genera determined in IC urine constituted only 2% of the total IC urine bacterial community.

In contrast to HF urine, there was a considerable reduction in total numbers of genera identified in IC urine (45 genera vs. 31 genera, respectively) (Additional file 1: Table S1). Additionally, the abundance of common genera was found to differ between IC patients and healthy females. The significant increase of *Lactobacillus* (*p* ≤ 0.05, *p* values from Metastats for both V1V2 and V6) in IC urine compared to HF urine again suggested a structural shift in the microbiota of IC patients. *Enterococcus, Atopobium, Proteus* and *Cronobacter* are 4 genera identified in IC urine that were not detected in our previous HF urine study, while a group of 17 genera were only associated with HF urine.

Sequences from 16 of the genera identified in the IC samples were further assigned to 22 different species (Additional file 3: Table S3). When comparing to our previous study, 13 of these species are already found in asymptomatic HF urine. However, nine of these species were not identified in our previous study, nor associated with IC according to literature.

### Variation between individual IC urine samples

**Figure 2:**
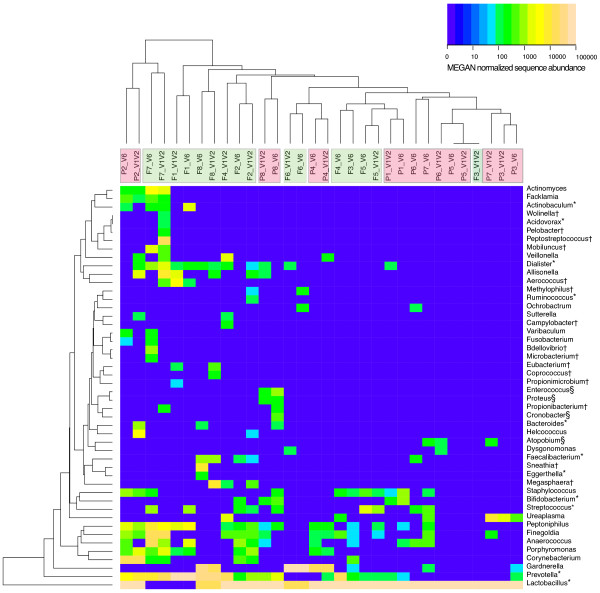
**Hierarchical clustering of urine microbiomes.** Heat map showing the relative abundance of bacterial genera across the urine samples. Genera are listed to the right. Subjects are listed at the top: interstitial cystitis (IC) samples denoted as P_number_V1V2 or V6, and healthy female (HF) urine samples as F_number_V1V2 or V6. Pink indicates IC urine, green HF urine. Color intensity of the heat map is directly proportional to log 10 scale of the abundance normalized sequence data as done by MEGAN V3.4. Taxa marked with (*) are genera that were significantly (*p* ≤ 0.05, *p* value from Metastats) different between the IC and HF urine microbiota. Genera marked with (†) and (§) are unique for HF urine sequences and IC urine sequences, respectively. Note that most of the IC urine samples are less complex than what is seen for HF urine samples.

A clustering analysis using taxonomical data from both IC and HF individual urine samples is shown in Figure 2. As previously demonstrated for HF urine (Siddiqui *et al*. 2011 [16]), variation between individuals was also evident for IC urine samples and a polymicrobial state was identified for all but one of the IC urine specimens. Although a clear clustering of samples from the two communities (IC and HF) was not apparent, we observed a narrower taxonomical range and reduced complexity in individual IC urine samples compared to the results from individual HF samples.

In all but two IC urine samples, *Lactobacillus* accounted for more than ~95% of the sequences for both V1V2 and V6 data. *Lactobacillus* was not only the most abundant genus, but also the most frequent genus among all IC urine specimens with its rRNA sequences present in all eight samples, in contrast to urine samples from HF (6/8). Sequences assigned to *Prevotella*, *Peptoniphilus* and *Anaerococcus* were also frequently detected (5/8), followed by *Staphylococcus* and *Finegoldia* (4/8), and *Gardnerella, Streptococcus* and *Dialister* (3/8) in IC urine. Including *Ureaplasma*, 7 genera were identified by reads belonging to 2 urine samples and another 15 genera were only detected in 1 out of the 8 samples.

### Species richness and diversity

**Figure 3:**
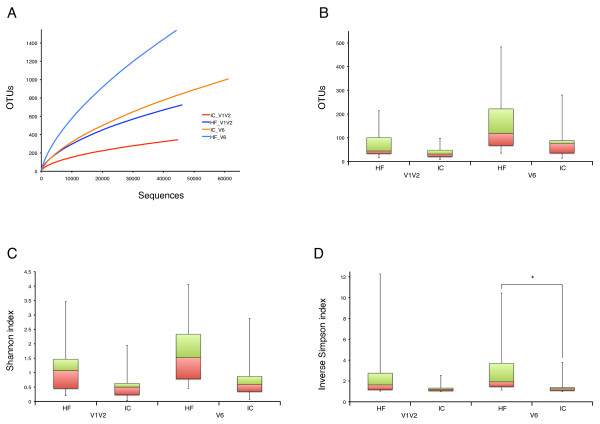
**Comparison of richness and diversity estimations of urine from interstitial cystitis (IC) patients and healthy females (HF). A**: Rarefaction curves depicting number of OTUs (at 3% genetic difference) as function of the total number of sequences for the combined sequence pool datasets for IC urine V1V2 and V6 (red and orange) and HF urine V1V2 and V6 (dark and light blue). The curves show a decreased estimate of species richness in the IC urine microbiome compared to the HF urine microbiome. **B**, **C**, and **D**: Box plots showing richness and diversity of 16S rDNA sequences. Boxes contain 50% of the data and have lines at the lower quartile (red), median and upper quartile (green) values. Ends of the whiskers mark the lowest and highest value. The plots show the results of a combined assessment of the eight urine samples in each HF and IC microbiome and with normalized numbers of sequences for OTU and Shannon index values (**B** and **C**). **B**: Observed OTU counts (at 3% genetic difference) of all urine samples taken from HF and IC, for both V1V2 and V6 datasets. **C** and **D**: Shannon index and inverse Simpson index at 3% sequence dissimilarity calculated to estimate diversity for both V1V2 and V6 datasets. Asterisks (*) indicate significant differences (Wilcox rank sum test: * *p* < 0.05). Note that a single sample (P2) in the IC community is the only outlier with the highest values for both richness and diversity (for both V1V2 and V6 analysis).

Estimation of species richness and diversity were calculated for the two combined V1V2 and V6 sequence pools (Table 1), as well as for single urine samples (Additional file 2: Table S2). At the species level, defined as OTUs at 3% genetic difference, 344 species for the V1V2 and 1,008 species for the V6 sequence datasets were estimated in the IC urine community. At a more conservative level, defined as OTUs at 6% dissimilarity level, 292 and 786 OTUs were estimated for V1V2 and V6, respectively. These numbers for richness are considerably lower than found in HF urine (Table 1 and Figure 3A). The number of OTUs at 3% difference for the individual samples for both IC and HF are indicated in box plots (Figure 3B) for both V1V2 and V6 analysis. In general, fewer number of OTU clusters were observed for IC individuals than that for HF individuals. Ecological diversity measured by Shannon and inverse Simpson indices also indicate lower diversity in IC urine in comparison to what was seen in urine from HF (Figure 3C and D). Specifically, a significant (*p* < 0.05) decrease in inverse Simpson index in IC patients compared to HF was found for the V6 analysis. Taken together, the results for both V1V2 and V6 support each other and confirm that the urine community is less diverse in IC patients than in HF individuals. However, the single IC outlier with high richness and diversity (Figure 3B-D) also clustered outside the IC group in the clustering analysis done using taxonomy data (Figure 2) showing that there is also potential for variation within the IC community.

**Figure 4:**
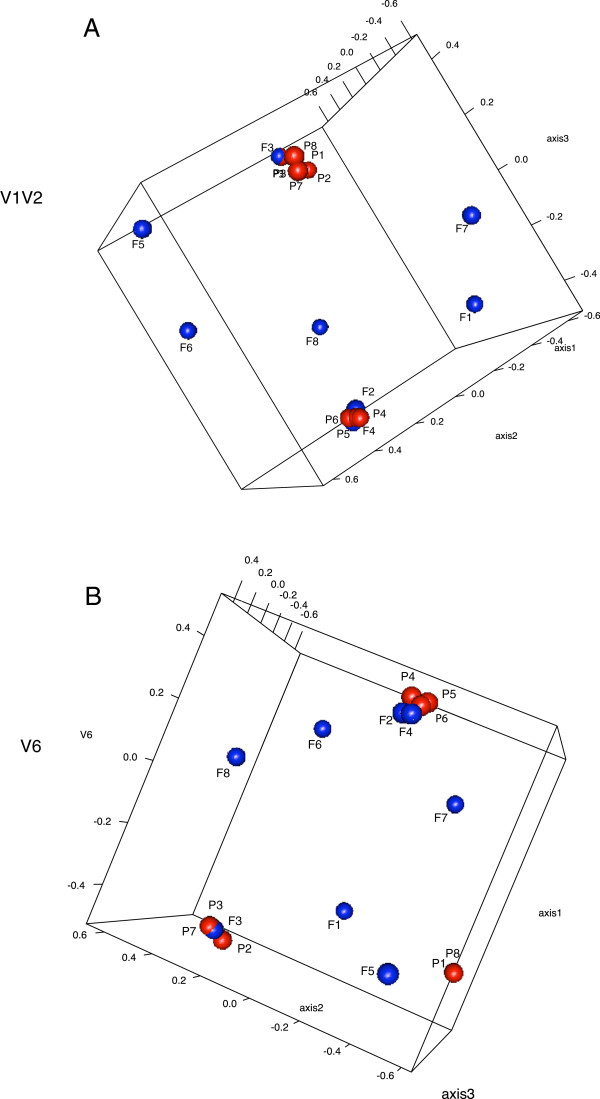
**OTU based clustering analysis of urine microbiomes.** Non-metric multidimensional scaling (NMDS) plots were generated based on θYC distances (0.03) between interstitial cystitis (IC) and healthy female (HF) microbiomes for both V1V2 (**A**) and V6 region (**B**). Red: IC patient samples; blue: HF samples.

The IC and HF urine also showed a degree of community similarity at 3% sequence dissimilarity level - about 12% and 9.5% of the total OTUs for V1V2 and V6, respectively, were present in both groups (Additional file 4: Figure S1). To further explore the relationships between the two communities, θYC distances, taking into account both community association and relative abundance, were calculated at 3% dissimilarity level for all samples. These distances for both V1V2 and V6 datasets were then visualized by NMDS plots; see Figure 4A and B. Although an overlap between the two communities is detected, HF urine samples were more dispersed than IC samples. A pattern of less variation between samples from IC patients than for HF samples was suggested. Weighted UniFrac hypothesis testing for θYC distances confirmed the significance (*p* < 0.001) of the differences observed in the community structure.

## Discussion

We have characterized the urine microbiota of IC patients using high throughput 454 pyrosesequencing of 16S rDNA amplicons. These results were compared to HF data from our previous study (Siddiqui *et al*. (2011) [16]). Our results did not reveal any single potential pathogenic bacterium common to all IC patients. However, important differences were detected between the IC and HF microbiota.

The use of primers for both V1V2 and V6 regions yielded complementary results for IC urine in line with the previous study of HF urine (Siddiqui *et al*. (2011) [16]), and thus maximized the detection of bacterial diversity.

Knowing that urine samples are at risk of contamination by bacterial flora of the female urogential system [34,35], mid-stream urine sampling was performed under guidance of an experienced urotherapy nurse. Suprapubic puncture was suggested as an alternative method, but the method was considered to be too invasive. Interestingly, comparing results from our previous microbiome study on female mid-stream urine (Siddiqui *et al*. 2011) [16] with recent results from suprapubic aspirate by Wolfe *et al*. (2012) [19], the major findings are the same; a strong indication that mid-stream urine will give comparable results in a urine microbiome analysis.

### A decrease in species richness in IC urine

A decrease in overall richness and ecological diversity (as indicated by rarefaction analysis, number of OTUs, Shannon index and inverse Simpson index estimations) of IC urine microbiota was detected in contrast to HF urine (Table 1 and Figure 3). In addition, the ß-diversity analysis (θYC distances between all urine samples) suggested that the microbiota of HF samples are more dissimilar from each other than the microbiota of IC individuals.

The taxonomical analysis indicated a shift in composition of urine microbiota of IC patients, with changes in bacterial groups spanning from genus to phyla level and a reduction in microbial complexity compared to HF. More importantly, a significant increase in *Lactobacillus* in IC patients was revealed. *Lactobacillus* was detected in every IC urine sample and a total sequence abundance of 92% in contrast to 57% in HF urine was observed. This shift was also clearly displayed both at the order and phylum level (*Lactobacillales* and *Firmicutes*, respectively). In contrast, *Prevotella,* - a genus belonging to the phylum *Bacteroidetes* (order *Bacteriodales*) – was present only at 1%, significantly lower than in HF urine, where it was previously reported as one of the major genera with an abundance of 19%. *Gardnerella*, another dominant genus in female urine, was present with the same frequency in IC urine but with a general lower abundance.

A reduction in bacterial diversity and shift in the microbiota as observed in this chronic inflammatory state has also been reported for other clinical conditions such as obesity, irritable bowel syndrome, and inflammatory bowel disease including Crohn’s disease [36-38].

### Bacteria associated with IC

Attempts to identify an infectious etiology for IC have not yet found any evidence for a specific pathogen. However, previous culture-dependent studies of samples from IC patients (i.e. bladder biopsy, midstream urine) have reported organisms such as *Gardnerella*, *Lactobacillus sp., Streptococcus* ssp.*, Escherichia coli*, *Proteus mirabilis*, *Corynebacterium* ssp., *Klebsiella* sp., *Enterococcus* sp*., Propionbacterium, Prevotella, Bacteroides* sp.*,* and *Peptostreptococcus*[6,9,39]. *Lactobacillus, Gardnerella* and *Streptococcus* were repeatedly detected in these studies and were also seen in our study. Haarala *et al*. (1999) [9] using culture techniques concluded that bacterial flora of midstream urine from patients with IC clearly differs from that of healthy women, in line with our findings. A study by Zhang *et al*. (2010) [15] suggested nanobacteria as a possible causative agent for IC. The two latter studies also reported a reduction in bacterial levels and urinary symptoms upon antibiotic treatment of the IC patients. The primer pairs both for V1V2 and V6 amplicons used in our study would be expected to amplify 16S rDNA regions of all of the organisms mentioned above. Nevertheless we did not identify *Klebsiella, E.coli*, *Peptostreptococcus* or nanobacteria in any of our IC urine samples.

Studies reporting results from culture-independent 16S rDNA PCR approaches on samples (i.e. bladder biopsy, midstream urine) from IC patients, have yielded somewhat conflicting results both in terms of positive PCRs and the resulting bacterial profiles [7,8,10,11,40]. While two of the reports [11,40] found no evidence of bacterial DNA in biopsy and urine specimens from IC patients, Dominique *et al*. (1995) [8] demonstrated bacterial DNA in bladder tissues in 29% of patients with IC. The 4 sequences retrieved showed homology to *E. coli* (2) and *Pseudomonas* (2), however neither of these bacteria was found in our study. Heritz *et al*. (1997) [10] also reported bacterial DNA in both biopsies and urines from IC patients (53% and 46%, respectively). They concluded that there is a difference in the bacterial profile between the patients and controls, and further suggested a link between one or more bacterial species and IC. However, in their study only 11 bacterial clones from 3 different IC patients were analyzed and the bacterial sequences were related to *E.coli, Abiotrophia defectivus, Veillonella* and *Rothia dentocariosa.* Except for *Veillonella*, these bacteria were not detected in our study. All these 4 previously reported studies used different primer sets (likely to explain some of the differences in the results) and classical cloning strategies (explaining the very few sequences analyzed). In contrast, our study represents the first 16S rDNA amplicon high throughput sequencing approach on IC urine, increasing both the sensitivity and resolution of the investigation.

### Significance of *Lactobacillus* in IC urine

*Lactobacillus* has not only been indicated or shown in IC urine samples from females (100% of the cases in this study and as shown by others [6,9,39]) but also demonstrated in IC urine from a male subject [41]. In our study we also detected a significant increase in abundance of this genus, considering its supposedly commensal presence in human urine from healthy subjects [16,18,19].

*Lactobacillus* is generally considered to be of low virulence, rarely causing infections in humans. It is best known for its presence in vaginal microflora, where it normally generates and maintains a physiological acidic environment, which prevents infections. Because of these properties, *Lactobacillus* has been used in probiotics, and is thought to prevent or even treat urinary tract infection (UTI) [42]. However, there are increasing indications that specific *Lactobacillus* spp are of pathogenic relevance and may be involved in urinary tract infections [43,44].

Many female patients with symptoms suggestive of UTI, but with culture-negative urines are often treated with antibacterial agents since their symptoms may be indistinguishable from those with a proven UTI [45]. It has been proposed that *Lactobacillus*, resistant to widely used antibiotics, may multiply during treatment, giving the genus an advantage over antibiotic-sensitive commensals, and allowing it to invade the proximal urethra and paraurethral tissues causing inflammatory changes [45]. This organism has also been related to the presence of UTI symptoms in otherwise culture-negative urines [43,44,46]. In a study by Maskell *et al*. (1983) [46] antibacterial treatment was withheld over the course of 2 years from symptomatic women with culture-negative urine. During the course of the study *Lactobacilli* (detected by special culture techniques) gradually disappeared from the urine of most of the patients who also became symptom free. A similar association of *Lactobacillu*s and urinary symptoms was reported by Darbro *et al*. (2009) [44]. Over a six-month period a female patient had constant symptoms and high counts of *Lactobacillus* spp (> 50,000 CFU/ml) in the urine; she became symptom-free with culture-negative urine after treatment with *Lactobacillus*-targeted medication. These results also suggest that a shift in the microbial community towards *Lactobacillus* in IC urine samples may be an important etiological factor for the severe symptoms reported by the patients. Since additional culture techniques such as 48 h incubation in an atmosphere containing 7% CO_2_ are needed for detection of *Lactobacillus*, this may be the reason why IC urine samples have not yet been associated with bacterial growth in routine clinical investigations. However, in our study this problem was overcome by a culture-independent approach.

## Conclusion

This investigation did not reveal any obvious putative causative bacterial agents of IC. However, the greater abundance of *Lactobacillus* in IC urine and its lower occurrence in HF urine is an important finding that requires further study to establish whether these microbial changes play a part in the development of IC. To this end, whole genome sequencing of *Lactobacillus* from IC patients may be a possible approach. Even if an increased presence of *Lactobacillus* is merely a secondary marker, understanding its IC associated genomics could aid in diagnosis and therapeutic assessment.

## Authors’ contribution

HS, AJN, SLJ and KSJ were involved in study design; HS processed the samples and carried out the molecular techniques. KL and HS performed the bioinformatics and taxonomic analysis. HS interpreted the data and authored the manuscript. All authors edited and commented on the paper and all authors read and approved the final manuscript.

## Supplementary Material

Additional file 1 **Table S1.** Differentially abundant taxa between interstitial cystitis (IC) and healthy female (HF) urine microbiota as estimated by Metastats (http://metastats.cbcb.umd.edu/). Click here for file

Additional file 2 **Table S2.** Sampling depth and biodiversity found by amplicon 454 pyrosequencing V1V2 and V6 region from eight interstitial cystitis (IC) and eight healthy female (HF) urine. Click here for file

Additional file 3 **Table S3.** Bacterial species identified in interstitial cystitis (IC) urine by 16S rDNA amplicon 454 pyrosequencing. Click here for file

Additional file 4 **Figure S1.** Venn diagrams for overlap between healthy female (HF) urine observed OTUs vs. interstitial cystitis (IC) urine OTUs, for both V1V2 (A) and V6 (B) region. The OTUs are calculated at 3% genetic sequence dissimilarity. Click here for file

## References

[B1] Payne CK Joyce GF Wise M Clemens JQ Interstitial cystitis and painful bladder syndrome J Urol 2007 177 6 2042 2049 10.1016/j.juro.2007.01.124 17509284

[B2] Abrams P Cardozo L Fall M Griffiths D Rosier P Ulmsten U van Kerrebroeck P Victor A Wein A The standardisation of terminology of lower urinary tract function: report from the Standardisation Sub-committee of the International Continence Society Neurourol Urodyn 2002 21 2 167 178 10.1002/nau.10052 11857671

[B3] Marinkovic SP Moldwin R Gillen LM Stanton SL The management of interstitial cystitis or painful bladder syndrome in women BMJ 2009 339 b2707 10.1136/bmj.b2707 19648180

[B4] Bouchelouche K Nordling J Recent developments in the management of interstitial cystitis Curr Opin Urol 2003 13 4 309 313 10.1097/00042307-200307000-00007 12811295

[B5] Hanno PM Diagnosis of interstitial cystitis Urol Clin North Am 1994 21 1 63 66 8284846

[B6] Keay S Schwalbe RS Trifillis AL Lovchik JC Jacobs S Warren JW A prospective study of microorganisms in urine and bladder biopsies from interstitial cystitis patients and controls Urology 1995 45 2 223 229 10.1016/0090-4295(95)80009-3 7855970

[B7] Keay S Zhang CO Baldwin BR Jacobs SC Warren JW Polymerase chain reaction amplification of bacterial 16S rRNA genes in interstitial cystitis and control patient bladder biopsies J Urol 1998 159 1 280 283 10.1016/S0022-5347(01)64082-5 9400495

[B8] Domingue GJ Ghoniem GM Bost KL Fermin C Human LG Dormant microbes in interstitial cystitis J Urol 1995 153 4 1321 1326 10.1016/S0022-5347(01)67594-3 7869536

[B9] Haarala M Kiilholma P Lehtonen OP Urinary bacterial flora of women with urethral syndrome and interstitial cystitis Gynecol Obstet Invest 1999 47 1 42 44 10.1159/000010060 9852391

[B10] Heritz DM Lacroix JM Batra SD Jarvi KA Beheshti B Mittelman MW Detection of eubacteria in interstitial cystitis by 16S rDNA amplification J Urol 1997 158 6 2291 2295 10.1016/S0022-5347(01)68237-5 9366378

[B11] Al-Hadithi HN Williams H Hart CA Frazer M Adams EJ Richmond DH Tincello DG Absence of bacterial and viral DNA in bladder biopsies from patients with interstitial cystitis/chronic pelvic pain syndrome J Urol 2005 174 1 151 154 10.1097/01.ju.0000161605.14804.a9 15947607

[B12] Warren JW Brown V Jacobs S Horne L Langenberg P Greenberg P Urinary tract infection and inflammation at onset of interstitial cystitis/painful bladder syndrome Urology 2008 71 6 1085 1090 10.1016/j.urology.2007.12.091 18538691

[B13] Burkhard FC Blick N Hochreiter WW Studer UE Urinary urgency and frequency, and chronic urethral and/or pelvic pain in females. Can doxycycline help? J Urol 2004 172 1 232 235 10.1097/01.ju.0000128698.93305.2e 15201781

[B14] Smith SD Wheeler MA Foster HE Jr Weiss RM Improvement in interstitial cystitis symptom scores during treatment with oral L-arginine J Urol 1997 158 3 Pt 1 703 708 9258064 10.1097/00005392-199709000-00005

[B15] Zhang QH Shen XC Zhou ZS Chen ZW Lu GS Song B Decreased nanobacteria levels and symptoms of nanobacteria-associated interstitial cystitis/painful bladder syndrome after tetracycline treatment Int Urogynecol J Pelvic Floor Dysfunct 2010 21 1 103 109 10.1007/s00192-009-0994-7 19760079

[B16] Siddiqui H Nederbragt AJ Lagesen K Jeansson SL Jakobsen KS Assessing diversity of the female urine microbiota by high throughput sequencing of 16S rDNA amplicons BMC Microbiol 2011 11 244 10.1186/1471-2180-11-244 22047020 PMC3228714

[B17] Nelson DE Van Der Pol B Dong Q Revanna KV Fan B Easwaran S Sodergren E Weinstock GM Diao L Fortenberry JD Characteristic male urine microbiomes associate with asymptomatic sexually transmitted infection PLoS One 2010 5 11 e14116 10.1371/journal.pone.0014116 21124791 PMC2991352

[B18] Dong Q Nelson DE Toh E Diao L Gao X Fortenberry JD Van Der Pol B The microbial communities in male first catch urine are highly similar to those in paired urethral swab specimens PLoS One 2011 6 5 e19709 10.1371/journal.pone.0019709 21603636 PMC3094389

[B19] Wolfe AJ Toh E Shibata N Rong R Kenton K Fitzgerald M Mueller ER Schreckenberger P Dong Q Nelson DE Evidence of uncultivated bacteria in the adult female bladder J Clin Microbiol 2012 50 4 1376 1383 10.1128/JCM.05852-11 22278835 PMC3318548

[B20] van de Merwe JP Nordling J Bouchelouche P Bouchelouche K Cervigni M Daha LK Elneil S Fall M Hohlbrugger G Irwin P Diagnostic criteria, classification, and nomenclature for painful bladder syndrome/interstitial cystitis: an ESSIC proposal Eur Urol 2008 53 1 60 67 10.1016/j.eururo.2007.09.019 17900797

[B21] Quince C Lanzen A Curtis TP Davenport RJ Hall N Head IM Read LF Sloan WT Accurate determination of microbial diversity from 454 pyrosequencing data Nat Methods 2009 6 9 639 641 10.1038/nmeth.1361 19668203

[B22] ESPRIThttp://www.biotech.ufl.edu/people/sun/esprit.html

[B23] MEtaGenome ANalyzerhttp://www-ab.informatik.uni-tuebingen.de/software/megan/welcome.html

[B24] Huson DH Auch AF Qi J Schuster SC MEGAN analysis of metagenomic data Genome Res 2007 17 3 377 386 Software freely available for academic purposes from http://www-ab.informatik.uni-tuebingen.de/software/megan 10.1101/gr.5969107 17255551 PMC1800929

[B25] Urich T Lanzen A Qi J Huson DH Schleper C Schuster SC Simultaneous assessment of soil microbial community structure and function through analysis of the meta-transcriptome PLoS One 2008 3 6 e2527 10.1371/journal.pone.0002527 18575584 PMC2424134

[B26] Metastatshttp://metastats.cbcb.umd.edu/

[B27] White JR Nagarajan N Pop M Statistical methods for detecting differentially abundant features in clinical metagenomic samples PLoS Comput Biol 2009 5 4 e1000352 10.1371/journal.pcbi.1000352 19360128 PMC2661018

[B28] Schloss PD Westcott SL Ryabin T Hall JR Hartmann M Hollister EB Lesniewski RA Oakley BB Parks DH Robinson CJ Introducing mothur: open-source, platform-independent, community-supported software for describing and comparing microbial communities Appl Environ Microbiol 2009 75 23 7537 7541 10.1128/AEM.01541-09 19801464 PMC2786419

[B29] Schloss PD Gevers D Westcott SL Reducing the effects of PCR amplification and sequencing artifacts on 16S rRNA-based studies PLoS One 2011 6 12 e27310 10.1371/journal.pone.0027310 22194782 PMC3237409

[B30] Huse SM Welch DM Morrison HG Sogin ML Ironing out the wrinkles in the rare biosphere through improved OTU clustering Environ Microbiol 2010 12 7 1889 1898 10.1111/j.1462-2920.2010.02193.x 20236171 PMC2909393

[B31] Lemos LN Fulthorpe RR Triplett EW Roesch LF Rethinking microbial diversity analysis in the high throughput sequencing era J Microbiol Methods 2011 86 1 42 51 10.1016/j.mimet.2011.03.014 21457733

[B32] Yue JC Clayton MK A similarity measure based on species proportions Commun Stat Theor M 2005 34 11 2123 2131 10.1080/STA-200066418

[B33] Lozupone CA Hamady M Kelley ST Knight R Quantitative and qualitative beta diversity measures lead to different insights into factors that structure microbial communities Appl Environ Microbiol 2007 73 5 1576 1585 10.1128/AEM.01996-06 17220268 PMC1828774

[B34] Jackson SR Dryden M Gillett P Kearney P Weatherall R A novel midstream urine-collection device reduces contamination rates in urine cultures amongst women BJU Int 2005 96 3 360 364 10.1111/j.1464-410X.2005.05631.x 16042730

[B35] Bekeris LG Jones BA Walsh MK Wagar EA Urine culture contamination: a College of American Pathologists Q-Probes study of 127 laboratories Arch Pathol Lab Med 2008 132 6 913 917 18517272 10.5858/2008-132-913-UCCACO

[B36] Ott SJ Musfeldt M Wenderoth DF Hampe J Brant O Folsch UR Timmis KN Schreiber S Reduction in diversity of the colonic mucosa associated bacterial microflora in patients with active inflammatory bowel disease Gut 2004 53 5 685 693 10.1136/gut.2003.025403 15082587 PMC1774050

[B37] Carroll IM Ringel-Kulka T Siddle JP Ringel Y Alterations in composition and diversity of the intestinal microbiota in patients with diarrhea-predominant irritable bowel syndrome Neurogastroenterol Motil 2012 24 6 521 e248 10.1111/j.1365-2982.2012.01891.x 22339879 PMC3975596

[B38] Turnbaugh PJ Hamady M Yatsunenko T Cantarel BL Duncan A Ley RE Sogin ML Jones WJ Roe BA Affourtit JP A core gut microbiome in obese and lean twins Nature 2009 457 7228 480 484 10.1038/nature07540 19043404 PMC2677729

[B39] Wilkins EG Payne SR Pead PJ Moss ST Maskell RM Interstitial cystitis and the urethral syndrome: a possible answer Br J Urol 1989 64 1 39 44 10.1111/j.1464-410X.1989.tb05519.x 2670041

[B40] Haarala M Jalava J Laato M Kiilholma P Nurmi M Alanen A Absence of bacterial DNA in the bladder of patients with interstitial cystitis J Urol 1996 156 5 1843 1845 10.1016/S0022-5347(01)65549-6 8863628

[B41] Lacroix JM Jarvic K Batrab SD Heritze DM Mittelman MW PCR-based technique for the detection of bacteria in semen and urine J Microbiol Methods 1996 26 1–2 61 71

[B42] Falagas ME Betsi GI Tokas T Athanasiou S Probiotics for prevention of recurrent urinary tract infections in women: a review of the evidence from microbiological and clinical studies Drugs 2006 66 9 1253 1261 10.2165/00003495-200666090-00007 16827601

[B43] Imirzalioglu C Hain T Chakraborty T Domann E Hidden pathogens uncovered: metagenomic analysis of urinary tract infections Andrologia 2008 40 2 66 71 10.1111/j.1439-0272.2007.00830.x 18336452

[B44] Darbro BW Petroelje BK Doern GV Lactobacillus delbrueckii as the cause of urinary tract infection J Clin Microbiol 2009 47 1 275 277 10.1128/JCM.01630-08 18987176 PMC2620876

[B45] Maskell RM The natural history of urinary tract infection in women Med Hypotheses 2010 74 5 802 806 10.1016/j.mehy.2009.12.011 20064694

[B46] Maskell R Pead L Sanderson RA Fastidious bacteria and the urethral syndrome: a 2-year clinical and bacteriological study of 51 women Lancet 1983 2 8362 1277 1280 6139621 10.1016/s0140-6736(83)91152-2

