# Supplementary material for: Alterations of microbiota in urine from women with interstitial cystitis
Source: BMC Microbiol. 2012 Sep 13;12:205. doi: 10.1186/1471-2180-12-205 (PMC3538702; doi:10.1186/1471-2180-12-205)
Supplement: Additional file 1 — Table S1. Differentially abundant taxa between interstitial cystitis (IC) and healthy female (HF) urine microbiota as estimated by Metastats (http://metastats.cbcb.umd.edu/). [file 1471-2180-12-205-S1.pdf]

**Table S2** Differentially abundant taxa between IC and healthy female urine microbiota as estimated by Metastats (http://metastats.cbcb.umd.edu)

| Taxon | IC urine abundance (n=8) | | Helalthy Female Urine abundance (n=8) | | Metastat p-value | |
|---|---|---|---|---|---|---|
| **Phyla** | **V1V2** | **V6** | **V1V2** | **V6** | **V1V2** | **V6** |
| **Spirochaetes** | 0.00000 ± 0 | 0.00000 ± 0 | 0.00023 ± 0.00023 | 0.00000 ± 0 | 0.001 | N/A |
| **Fusobacteria** | 0.00000 ± 0 | 0.00008 ± 0.00008 | 0.04300 ± 0.04139 | 0.01394 ± 0.01350 | 0.001 | 0.194 |
| **Synergistetes** | 0.00000 ± 0 | 0.00000 ± 0 | 0.00017 ± 0.00017 | 0.00000 ± 0 | 0.001 | N/A |
| **Fibrobacteres** | 0.00000 ± 0 | 0.00000 ± 0 | 0.00019 ± 0.00019 | 0.00000 ± 0 | 0.001 | N/A |
| **Acidobacteria** | 0.00000 ± 0 | 0.00000 ± 0 | 0.00023 ± 0.00023 | 0.00047 ± 0.00047 | 0.001 | 0.001 |
| **Bacteroidetes** | 0.01709 ± 0.01507 | 0.01114 ± 0.00767 | 0.19597 ± 0.10968 | 0.16292 ± 0.08882 | 0.045 | 0.045 |
| **Firmicutes** | 0.93397 ± 0.03406 | 0.92278 ± 0.04318 | 0.62354 ± 0.13265 | 0.65640 ± 0.12550 | 0.042 | 0.057 |
| Tenericutes | 0.01027 ± 0.00739 | 0.00113 ± 0.00076 | 0.00229 ± 0.00229 | 0.00025 ± 0.00025 | 0.202 | 0.204 |
| Proteobacteria | 0.00089 ± 0.00046 | 0.00285 ± 0.00196 | 0.01922 ± 0.01766 | 0.01672 ± 0.01549 | 0.317 | 0.644 |
| Actinobacteria | 0.03776 ± 0.02349 | 0.06195 ± 0.03827 | 0.11512 ± 0.09155 | 0.14904 ± 0.09922 | 0.638 | 0.426 |
| Chloroflexi | 0.00000 ± 0 | 0.00000 ± 0 | 0.00000 ± 0 | 0.00021 ± 0.00021 | N/A | 0.001 |
| **Nitrospirae** | 0.00000 ± 0 | 0.00005 ± 0.00005 | 0.00000 ± 0 | 0.00000 ± 0 | N/A | 0.001 |
| **Order** | **V1V2** | **V6** | **V1V2** | **V6** | **V1V2** | **V6** |
| **Xanthomonadales** | 0.00000 ± 0 | 0.00000 ± 0 | 0.00020 ± 0.00020 | 0.00058 ± 0.00058 | 0.001 | 0.001 |
| **Spirochaetales** | 0.00000 ± 0 | 0.00000 ± 0 | 0.00024 ± 0.00024 | 0.00000 ± 0 | 0.001 | N/A |
| **Sphingobacteriales** | 0.00000 ± 0 | 0.00014 ±0.00014 | 0.00024 ± 0.00024 | 0.00094 ± 0.00094 | 0.001 | 0.476 |
| Rhizobiales | 0.00000 ± 0 | 0.00014 ±0.00014 | 0.00000 ± 0 | 0.00045 ±0.00029 | N/A | 0.221 |
| Pasteurellales | 0.00036 ± 0.00036 | 0.00000 ± 0 | 0.00008 ± 0.00008 | 0.00000 ± 0 | 0.511 | N/A |
| **Neisseriales** | 0.00000 ± 0 | 0.00023 ± 0.00023 | 0.00049 ± 0.00049 | 0.00018 ± 0.00018 | 0.001 | 0.443 |
| **Myxococcales** | 0.00000 ± 0 | 0.00000 ± 0 | 0.00000 ± 0 | 0.00054 ± 0.00054 | N/A | 0.001 |
| Mycoplasmatales | 0.01027 ± 0.00739 | 0.00113 ± 0.0008 | 0.00229 ± 0.00229 | 0.00026 ± 0.00026 | 0.194 | 0.217 |
| **Methylophilales** | 0.00000 ± 0 | 0.00012 ± 0.00012 | 0.00033 ± 0.0002 | 0.00076 ± 0.0005 | 0.001 | 0.221 |
| **Lactobacillales** | 0.91971 ± 0.04387 | 0.90650 ± 0.05126 | 0.52102 ± 0.15793 | 0.53541 ± 0.15647 | 0.041 | 0.053 |
| **Fusobacteriales** | 0.00000 ± 0 | 0.00008 ± 0.00008 | 0.04158 ± 0.04158 | 0.01421 ± 0.01375 | 0.001 | 0.183 |
| **Enterobacteriales** | 0.00035 ± 0.00035 | 0.00179 ± 0.00179 | 0.00000 ± 0 | 0.00062 ± 0.00062 | 0.001 | 0.460 |
| **Desulfuromonadale** | 0.00000 ± 0 | 0.00000 ± 0 | 0.0004 ± 0.00040 | 0.00000 ± 0 | 0.001 | N/A |
| **Coriobacteriales** | 0.00057 ± 0.00039 | 0.00036 ± 0.00036 | 0.00048 ± 0.00032 | 0.00028 ± 0.00028 | 0.706 | 0.496 |
| Clostridiales | 0.01239 ± 0.0098 | 0.01268 ± 0.00824 | 0.10448 ± 0.07051 | 0.12014 ± 0.08018 | 0.175 | 0.111 |
| **Campylobacterales** | 0.00000 ± 0 | 0.00000 ± 0 | 0.00215 ± 0.01640 | 0.00921 ± 0.00921 | 0.001 | 0.001 |
| **Burkholderiales** | 0.00018 ± 0.00018 | 0.00000 ± 0 | 0.00063 ± 0.0005 | 0.00138 ± 0.00921 | 0.483 | 0.001 |
| Bifidobacteriales | 0.01840 ± 0.01776 | 0.0301 ± 0.02797 | 0.10829 ± 0.09246 | 0.13671 ± 0.10128 | 0.646 | 0.417 |
| **Bdellovibrionales** | 0.00000 ± 0 | 0.00000 ± 0 | 0.00000 ± 0 | 0.00098 ± 0.00098 | N/A | 0.001 |
| **Bacteroidales** | 0.01711 ± 0.01508 | 0.01092 ± 0.00781 | 0.19496 ± 0.10973 | 0.16243 ± 0.09076 | 0.029 | 0.045 |
| Bacillales | 0.00183 ± 0.00096 | 0.00349 ± 0.00188 | 0.00074 ± 0.00043 | 0.00132 ± 0.00059 | 0.337 | 0.362 |
| Actinomycetales | 0.01881 ± 0.01849 | 0.03198 ± 0.03144 | 0.00652 ± 0.00614 | 0.01343 ± 0.00958 | 0.757 | 0.967 |
| **Genenra** | **V1V2** | **V6** | **V1V2** | **V6** | **V1V2** | **V6** |
| **Proteus** | 0.00024 ± 0.00024 | 0.00024 ± 0.00024 | 0.00000 ± 0 | 0.00000 ± 0 | 0.001 | 0.001 |
| **Pelobacter** | 0.00000 ± 0 | 0.00000 ± 0 | 0.00051 ± 0.00051 | 0.00000 ± 0 | 0.001 | N/A |
| **Campylobacter** | 0.00000 ± 0 | 0.00000 ± 0 | 0.00025 ± 0.00025 | 0.00000 ± 0 | 0.001 | N/A |
| **Wolinella** | 0.00000 ± 0 | 0.00000 ± 0 | 0.00026 ± 0.00026 | 0.00000 ± 0 | 0.001 | N/A |
| **Acidovorax** | 0.00000 ± 0 | 0.00000 ± 0 | 0.00026 ± 0.00026 | 0.00000 ± 0 | 0.001 | N/A |
| **Methylophilus** | 0.00000 ± 0 | 0.00000 ± 0 | 0.00008 ± 0.00008 | 0.00000 ± 0 | 0.001 | N/A |
| **Bacteroides** | 0.00092 ± 0.00092 | 0.00018 ± 0.00018 | 0.00000 ± 0 | 0.00018 ± 0.00018 | 0.001 | 0.472 |
| **Streptococcus** | 0.00000 ± 0 | 0.00095 ± 0.00051 | 0.00149 ± 0.00133 | 0.00530 ± 0.00245 | 0.001 | 0.131 |
| **Enterococcus** | 0.00032 ± 0.00032 | 0.00114 ± 0.00114 | 0.00000 ± 0 | 0.00000 ± 0 | 0.001 | 0.001 |
| **Aerococcus** | 0.00000 ± 0 | 0.00000 ± 0 | 0.00539 ± 0.00439 | 0.00012 ± 0.00012 | 0.001 | 0.001 |
| **Coprococcus** | 0.00000 ± 0 | 0.00000 ± 0 | 0.00039 ± 0.00039 | 0.00000 ± 0 | 0.001 | N/A |
| **Megasphaera** | 0.00000 ± 0 | 0.00000 ± 0 | 0.02339 ± 0.02223 | 0.00000 ± 0 | 0.001 | N/A |
| **Peptostreptococcus** | 0.00000 ± 0 | 0.00000 ± 0 | 0.02516 ± 0.02516 | 0.00000 ± 0 | 0.001 | N/A |
| **Eubacterium** | 0.00000 ± 0 | 0.00000 ± 0 | 0.00079 ± 0.00065 | 0.00000 ± 0 | 0.001 | N/A |
| **Ruminococcus** | 0.00000 ± 0 | 0.00000 ± 0 | 0.00012 ± 0.00012 | 0.00000 ± 0 | 0.001 | N/A |
| **Faecalibacterium** | 0.00000 ± 0 | 0.00026 ± 0.00026 | 0.00187 ± 0.00177 | 0.00205 ± 0.00189 | 0.001 | 0.438 |
| **Actinobaculum** | 0.00000 ± 0 | 0.00016 ± 0.00016 | 0.00056 ± 0.00056 | 0.00269 ± 0.00219 | 0.001 | 0.210 |
| **Mobiluncus** | 0.00000 ± 0 | 0.00000 ± 0 | 0.00118 ± 0.00118 | 0.00275 ± 0.00275 | 0.001 | 0.001 |
| **Propionibacterium** | 0.00000 ± 0 | 0.00024 ± 0.00024 | 0.00041 ± 0.0004 | 0.00000 ± 0 | 0.001 | 0.001 |
| **Propionimicrobium** | 0.00000 ± 0 | 0.00000 ± 0 | 0.00007 ± 0.00007 | 0.00000 ± 0 | 0.001 | N/A |
| **Bifidobacterium** | 0.00057 ± 0.00037 | 0.00169 ± 0.00115 | 0.00000 ± 0 | 0.00037 ± 0.00037 | 0.001 | 0.193 |
| **Atopobium** | 0.00058 ± 0.00039 | 0.00036 ± 0.00036 | 0.00000 ± 0 | 0.00000 ± 0 | 0.001 | 0.001 |
| **Lactobacillus** | 0.92386 ± 0.04189 | 0.91319 ± 0.04710 | 0.52676 ± 0.15829 | 0.52209 ± 0.16405 | 0.036 | 0.050 |
| **Prevotella** | 0.01421 ± 0.01261 | 0.00913 ±0.00703 | 0.21109 ± 0.11331 | 0.18262 ± 0.10803 | 0.049 | 0.043 |
| **Dialister** | 0.00048 ± 0.00028 | 0.00000 ± 0 | 0.00465 ± 0.00351 | 0.00267 ± 0.00193 | 0.081 | 0.001 |
| Anaerococcus | 0.00009 ± 0.00009 | 0.00216 ± 0.00091 | 0.00169 ± 0.00142 | 0.04751 ± 0.04376 | 0.198 | 0.331 |
| Ureaplasma | 0.01030 ± 0.00742 | 0.00114 ± 0.00077 | 0.00231 ± 0.00231 | 0.00026 ± 0.00026 | 0.200 | 0.217 |
| Gardnerella | 0.01788 ± 0.01788 | 0.02777 ± 0.02754 | 0.11642± 0.09295 | 0.14113 ± 0.10249 | 0.208 | 0.400 |
| Peptoniphilus | 0.00139 ± 0.00093 | 0.00334 ± 0.00247 | 0.01245 ± 0.00931 | 0.02174 ± 0.01457 | 0.267 | 0.388 |
| Allisonella | 0.00214 ± 0.00201 | 0.00000 ± 0 | 0.01576 ± 0.01288 | 0.00000 ± 0 | 0.271 | N/A |
| Veillonella | 0.00051 ± 0.00033 | 0.00000 ± 0 | 0.00298 ± 0.00223 | 0.00000 ± 0 | 0.351 | N/A |
| Finegoldia | 0.00105 ± 0.00054 | 0.00178 ± 0.00114 | 0.03175 ± 0.03049 | 0.03799 ± 0.03652 | 0.402 | 0.524 |
| Dysgonomonas | 0.00018 ± 0.00018 | 0.00000 ± 0 | 0.00024 ± 0.00024 | 0.00000 ± 0 | 0.457 | N/A |
| Facklamia | 0.00017 ± 0.00017 | 0.00070 ±0.00070 | 0.00056 ± 0.00056 | 0.00072 ± 0.00072 | 0.458 | 0.468 |
| Actinomyces | 0.00028 ± 0.00028 | 0.00037 ± 0.00037 | 0.00405 ± 0.00405 | 0.00580 ± 0.00580 | 0.458 | 0.468 |
| Staphylococcus | 0.00090 ± 0.00083 | 0.00362 ± 0.00195 | 0.00018 ± 0.00018 | 0.00144 ± 0.00064 | 0.465 | 0.388 |
| Sutterella | 0.00019 ± 0.00019 | 0.00000 ± 0 | 0.00010 ± 0.00010 | 0.00000 ± 0 | 0.482 | N/A |
| Helcococcus | 0.00405 ± 0.00405 | 0.00000 ± 0 | 0.00010 ± 0.00010 | 0.00000 ± 0 | 0.487 | N/A |
| Porphyromonas | 0.00229 ± 0.00209 | 0.00115 ± 0.0008 | 0.00588 ± 0.00452 | 0.00153 ± 0.00086 | 0.587 | 0.585 |
| Corynebacterium | 0.01740 ± 0.01740 | 0.02948 ± 0.02948 | 0.00077 ± 0.00054 | 0.00161 ± 0.00079 | 0.745 | 0.859 |
| **Cronobacter** | 0.00000 ± 0 | 0.0004 ± 0.0004 | 0.00000 ± 0 | 0.00000 ± 0 | N/A | 0.001 |
| **Bdellovibrio** | 0.00000 ± 0 | 0.00000 ± 0 | 0.00000 ± 0 | 0.00132 ± 0.00132 | N/A | 0.001 |
| **Methylotenera** | 0.00000 ± 0 | 0.00000 ± 0 | 0.00000 ± 0 | 0.00023 ± 0.00023 | N/A | 0.001 |
| **Sneathia** | 0.00000 ± 0 | 0.00000 ± 0 | 0.00000 ± 0 | 0.01584 ± 0.01584 | N/A | 0.001 |
| **Eggerthella** | 0.00000 ± 0 | 0.00000 ± 0 | 0.00000 ± 0 | 0.00034 ± 0.00034 | N/A | 0.001 |
| Ochrobactrum | 0.00000 ± 0 | 0.00014 ± 0.00014 | 0.00000 ± 0 | 0.00023 ± 0.00023 | N/A | 0.464 |
| Fusobacterium | 0.00000 ± 0 | 0.00009 ± 0.00009 | 0.00000 ± 0 | 0.00054 ± 0.00054 | N/A | 0.468 |
| Varibaculum | 0.00000 ± 0 | 0.00024 ± 0.00024 | 0.00000 ± 0 | 0.00042 ± 0.00042 | N/A | 0.468 |
| **Microbacterium** | 0.00000 ± 0 | 0.00000 ± 0 | 0.00000 ± 0 | 0.00046 ± 0.00046 | N/A | 0.001 |

All phylum, order and genus levels (mean ± std.err.) are listed.

Taxa in bold are those who are significantly ($p \leq 0.05$ ) diffierent between the two communities, by either V1V2 or V6 method.

N/A : no p-value available
