# Supplementary material for: Alterations of microbiota in urine from women with interstitial cystitis
Source: BMC Microbiol. 2012 Sep 13;12:205. doi: 10.1186/1471-2180-12-205 (PMC3538702; doi:10.1186/1471-2180-12-205)
Supplement: Additional file 2 — Table S2. Sampling depth and biodiversity found by amplicon 454 pyrosequencing V1V2 and V6 region from eight interstitial cystitis (IC) and eight healthy female (HF) urine. [file 1471-2180-12-205-S2.pdf]

Table S1 Sampling depth and biodiversity found by amplicon 454 pyrosequencing V1V2 and V6 region from eight interstitial cystitis (IC) and eight healthy female (HF) urine

		Preprocessing				*Taxonomy analysis*		*OTU and Diversity indices*									
		Total reads	Length cut off [1]	Denoised[2]	Cleaned[3]	Phyla[4]	Genera[4]	Silva 16S alignment[5]	Unique OTUs	OTUs[6] (3%)	Chao1[7] (3%)	Chao1 LCI95	Caho1 HCI95	OTUs[6] (6%)	Normalized OTU[8] (3%)	Normalized Shannon index[9] (3%)	Inverse Simpson index[10] (3%)
P1	V1V2	16279	9516	9515	9506	3	5	9417	61	47	182	92	454	40	23	0.56	1.29
	V6	21026	11812	11804	11800	3	6	11705	145	110	353	231	597	89	41	0.39	1.11
P2	V1V2	10210	4754	4754	4753	4	15	4738	177	124	254	184	404	99	97	1.95	2.53
	V6	17644	8057	7955	7954	4	13	7915	710	507	1228	1021	1219	392	280	2.87	3.79
P3	V1V2	4913	3036	3036	3035	2	2	3020	14	11	19	12	53	9	10	0.17	1.06
	V6	19776	14611	14611	14602	5	4	14575	62	54	132	85	248	49	18	0.21	1.06
P4	V1V2	8198	5604	5603	5593	3	7	5370	93	70	152	105	260	64	45	0.69	1.41
	V6	8308	5753	5741	5730	4	6	5221	136	122	273	205	396	110	76	0.92	1.57
P5	V1V2	8317	6162	6162	6162	1	1	6107	15	13	22	15	53	12	7	0.03	1
	V6	7008	5463	5463	5463	1	1	5460	25	24	71	38	75	22	14	0.07	1
P6	V1V2	8677	4666	4663	4564	3	3	3518	86	75	247	150	466	70	53	0.61	1.21
	V6	8658	5746	5728	5617	3	5	5492	198	168	460	336	675	153	108	0.87	1.31
P7	V1V2	7650	5649	5649	5649	4	4	5632	74	52	134	83	270	47	33	0.43	1.17
	V6	8560	5976	5966	5966	4	9	5956	180	120	200	162	273	90	81	0.57	1.13
P8	V1V2	9967	6885	6885	6876	4	10	6792	60	45	59	49	87	43	28	0.25	1.06
	V6	7740	4907	4905	4900	4	11	4846	125	99	175	135	259	82	77	0.65	1.18
F1	V1V2	14579	8479	8479	8476	4	8	8455	61	48	71	55	118	44	29	0.51	1.19
	V6	18362	8039	7977	7969	4	8	7951	389	275	638	501	856	217	159	2	2.3
F2	V1V2	12629	8416	8416	8353	6	15	7557	290	234	485	390	638	201	119	1.28	1.56
	V6	6565	4752	4703	4682	3	10	4611	325	266	571	466	732	225	199	1.6	1.73
F3	V1V2	4305	2721	2721	2720	1	1	2710	19	16	25	17	60	14	16	0.21	1.06
	V6	17474	13066	13064	13060	3	8	13043	119	93	203	147	317	74	35	0.5	1.25
F4	V1V2	9877	6253	6253	6242	4	10	6074	81	59	96	72	161	51	42	1.27	2.04
	V6	5005	3467	3461	3459	4	5	3450	105	87	232	155	396	77	79	1.45	2.11
F5	V1V2	12645	10116	10116	10109	3	6	10085	98	71	113	89	170	56	35	0.33	1.08
	V6	6586	5074	5057	5053	3	4	5049	85	66	195	119	289	58	47	0.45	1.13
F6	V1V2	8216	4428	4427	4361	3	4	3221	74	68	324	175	678	67	46	0.87	1.68
	V6	5692	3047	3031	2988	4	4	2765	88	73	293	168	580	68	74	0.88	1.53
F7	V1V2	7861	3967	3967	3711	8	19	3425	248	293	367	321	443	220	214	3.46	12.28
	V6	6986	3495	3432	3138	7	17	3094	577	500	1375	1133	1709	449	483	4.06	10.45
F8	V1V2	8234	4481	4481	4480	4	9	4474	164	130	292	218	427	116	94	2.05	4.88
	V6	7397	4442	4411	4411	4	8	4183	518	379	1043	834	1347	304	290	3.31	7.89

[1]Length cutoff at minimum 218 nt for V1V2 and 235 nt for V6 reads.

[2]Total number of sequences after processing the dataset through the Pyronoise [21].

[3]The number of reads per dataset after removal of sequences that could be from the same source as those in the contamination control dataset as described in Siddiqui *et al*. (2011) [16].

[4]Number of phyla and genera are based on taxonomic classification by MEGAN V3.4 [23, 24].

[5]The number of total reads after Silva 16S alignment as recommended by MOTHUR [29].

[6]OTUs: Operational Taxonomic Units at 3% or 6% nucleotide difference.

[7]Chao1 is an estimator of the minimum richness and is based on the number of rare OTUs (singletons and doublets) within a sample.

[8]The number of OTUs after normalization of the number of sequences (as described in Methods).

[9]The Shannon index combines estimates of richness (total number of OTUs) and evenness (relative abundance) The values listed are after normalization of the number of sequences (as described in Methods).

[10]Inverse Simpson index (1/D) is an indication of the richness in a community with uniform evenness that would have the same level of diversity. It takes into account the number of OTUs present, as well as the abundance of each OTU.
