# Supplementary material for: Alterations of microbiota in urine from women with interstitial cystitis
Source: BMC Microbiol. 2012 Sep 13;12:205. doi: 10.1186/1471-2180-12-205 (PMC3538702; doi:10.1186/1471-2180-12-205)
Supplement: Additional file 3 — Table S3. Bacterial species identified in interstitial cystitis (IC) urine by 16S rDNA amplicon 454 pyrosequencing. [file 1471-2180-12-205-S3.pdf]

Table S3 Bacteria genus and species identified in interstitial cystitis patient urine by 16S rDNA amplicon 454 pyrosequencing

Genus	Species
Actinobaculum	
Actinomyces	*Actinomyces europaeus**
Allisonella	*uncultured Allisonella sp.*
Anaerococcus	*Anaerococcus hydrogenalis**
	uncultured Anaerococcus sp.
Atopobium	
Bacteroides	*Bacteroides coagulans**
Bifidobacterium	
Corynebacterium	*Corynebacterium pseudogenitalium*
	*Corynebacterium riegelii**
Cronobacter	*Cronobacter sakazakii**
Dialister	*Dialister pneumosintes**
Dysgonomonas	
Enterococcus	
Facklamia	*Facklamia hominis*
Faecalibacterium	*Faecalibacterium prausnitzii*
Finegoldia	*Finegoldia magna*
Fusobacterium	
Gardnerella	
Helcococcus	
Lactobacillus	*Lactobacillus coleohominis*
	Lactobacillus crispatus
Ochrobactrum	
Peptoniphilus	
Porphyromonas	*Porphyromonas somerae*
	*Porphyromonas uenonis**
	uncultured Porphyromonas sp.
Prevotella	*Prevotella disiens*
Propionibacterium	*Propionibacterium acnes**
Proteus	
Staphylococcus	
Streptococcus	*Streptococcus agalactiae**
Sutterella	
Ureaplasma	
Varibaculum	
Veillonella	*uncultured Veillonella sp.*
Family env samples	*uncultured Clostridiales bacterium*

* Species not detected in healthy female urine.
