# Supplementary material for: Alterations of microbiota in urine from women with interstitial cystitis
Source: BMC Microbiol. 2012 Sep 13;12:205. doi: 10.1186/1471-2180-12-205 (PMC3538702; doi:10.1186/1471-2180-12-205)
Supplement: Additional file 4 — Figure S1. Venn diagrams for overlap between healthy female (HF) urine observed OTUs vs. interstitial cystitis (IC) urine OTUs, for both V1V2 (A) and V6 (B) region. The OTUs are calculated at 3% genetic sequence dissimilarity. [file 1471-2180-12-205-S4.pdf]

A

Venn Diagram: OTUs at distance 0.03 for V1V2 16S rDNA

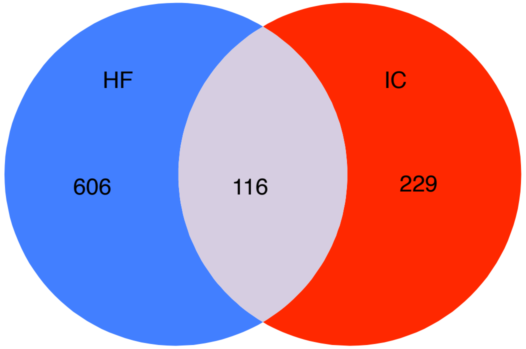

The number of OTUs (0.03) for V1V2 in HF(healthy female) urine is 722
The number of OTUs (0.03) for V1V2 in IC (Interstitial cystitis) urine is 345
The number of shared OTUs (0.03) for V1V2 between HF and IC urine is 116
Percentage of OTUs (0.03) for V1V2 that are shared in HF and IC urine is 12.1977
The total richness for V1V2 for both HF and IC is 951

B

Venn Diagram: OTUs at distance 0.03 for V6 16S rDNA

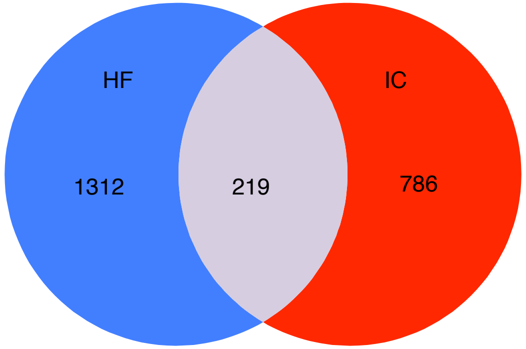

The number of OTUs (0.03) for V6 in HF(healthy female) urine is1531
The number of OTUs (0.03) for V6 in IC (Interstitial cystitis) urine is 1005
The number of shared OTUs (0.03) for V6 between HF and IC urine is 219
Percentage of OTUs (0.03) for V6 that are shared in HF and IC urine is 9.45188
The total richness for V6 for both HF and IC is 2317
